# Mechanistic modeling as an explanatory tool for clinical treatment of chronic catatonia

**DOI:** 10.3389/fphar.2022.1025417

**Published:** 2022-11-09

**Authors:** Patrick D. Roberts, James Conour

**Affiliations:** ^1^ Amazon Web Services, Portland, OR, United States; ^2^ Cascadia Behavioral Healthcare, Portland, OR, United States

**Keywords:** schizophrenia, Bush-Francis Catatonia Rating Scale, quantitative systems pharmacology, antipsychotic, benzodiazepine, lamotrigine, Wilson-Cowan

## Abstract

Mathematical modeling of neural systems is an effective means to integrate complex information about the brain into a numerical tool that can help explain observations. However, the use of neural models to inform clinical decisions has been limited. In this study, we use a simple model of brain circuitry, the Wilson-Cowan model, to predict changes in a clinical measure for catatonia, the Bush-Francis Catatonia Rating Scale, for use in clinical treatment of schizophrenia. This computational tool can then be used to better understand mechanisms of action for pharmaceutical treatments, and to fine-tune dosage in individual cases. We present the conditions of clinical care for a residential patient cohort, and describe methods for synthesizing data to demonstrated the functioning of the model. We then show that the model can be used to explain effect sizes of treatments and estimate outcomes for combinations of medications. We conclude with a demonstration of how this model could be personalized for individual patients to inform ongoing treatment protocols.

## 1 Introduction

The treatment of severe and persistent mental illness has been a central challenge for psychiatry. Individuals with the most debilitating forms of schizophrenia often derive limited benefit from medications. Additionally, the efficacy of pharmacologic treatments can be highly variable. A full response to a medical intervention may take weeks or months to materialize. Moreover, it can be difficult to accurately assess the impact of a specific medication. These challenges are compounded by the inconsistent history of care for many psychiatric patients and the significant amounts of polypharmacy they have been prescribed. Technical tools offer a promising augmentation to a psychiatrist’s experience to design treatment plans and may help reduce the inconsistencies and refine treatment for individual cases.

Catatonia manifests as a cluster of symptoms including rituals, repetitive movements, perseveration, and withdrawal (Northoff 2002). There is common co-morbidity with both psychiatric and medical illnesses (Bhati et al. 2007) and catatonia is often not recognized in its chronic form because it can present subtly and idiosyncratically (Penland et al. 2006). In individuals with treatment resistant schizophrenia, chronic catatonic may be quite common, and direct treatment of catatonic symptoms improves cognition (Wilcox and Reid Duffy 2015; Ungvari et al. 2005). For this reason, we have focussed on using the Bush-Francis Catatonia Rating Scale (BFCRS) (Bush et al. 1996) as a measure of symptoms and then model pharmacological mechanisms that explain how medications alleviate catatonic symptoms.

The data in this study is based on a cohort of schizophrenia patients admitted to Cascadia Behavioral Healthcare for residential care. The clinical practice in treating these patients has been to introduce a minimal set of medications with a known effect of reducing psychiatric symptoms. For patients admitted with a diagnosis of schizophrenia, antipsychotic medication was transitioned to clozapine (if possible), and augmented lamotrigine and a benzodiazepine based on functional status and safety. Lamotrigine has been previously observed to reduce symptoms in combination with clozapine (Gray and Risch, 2009; Tiihonen et al. 2003). Benzodiazepines have shown a strong therapeutic efficacy in reducing catatonia symptoms (Rosebush and Mazurek (2010); Northoff et al. 2004) and are considered a first-line treatment for acute or chronic catatonia (Ungvari et al. 2005). A significant reduction in catatonic symptoms, as measured by BFCRS, was observed in the clinic with this treatment along with a corresponding improvement in psychiatric symptoms. However, a mechanistic understanding of the action of this combination is desirable to improve treatments and seek new strategies for psychiatric disease maintenance.

### 1.1 Modeling as an explanatory tool

Physiological modeling of pharmacological systems can provide insight into mechanisms of therapeutic treatments by coupling molecular action to observable function. Explanatory models require a balance between biological detail and conceptional simplicity to express how specific treatments result in observed functional changes. The psychomotor abnormalities observed in catatonia can be conceptualized as a seizing of motor patterns on a time scale long enough to result in the clinical observations such as posturing and repetitive movements. Clinical and imaging studies have suggested that the physiological basis of catatonia symptoms are cortical in origin (Northoff et al. 2004; Hirjak et al. 2019) resulting from an over-excitation of circuitry and under-gating of movement termination. The effective treatments also support the concept of an imbalance of inhibition and excitation in cortical structures because targets of lamotrigine reduce pyramidal cell excitation (Poolos et al. 2002; Xie et al. 1995), and benzodiazepines increase inhibition (Miller et al. 1987).

**FIGURE 1:**
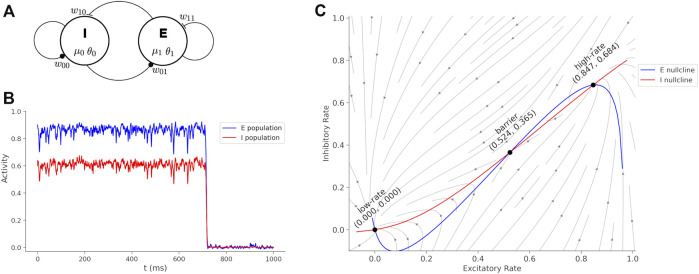
Wilson-Cowan model and dynamics. **(A)** Wilson-Cowan circuit with an inhibitory (I) and excitatory (E) neuron population. The model parameters associated with each circuit element are show. **(B)** Sustained activity eventually decays due to random perturbations drawn from a normal distribution with mean = 0 and standard deviation = 0.19. If the boundary is too high, then the sustained burst continues indefinitely. Treatments reduce the boundary between the states and transitions become more fluid and interpreted as a reduction of catatonia symptoms. **(C)** Phase plane of the Wilson-Cowan model with trajectories, nullclines and fixed points labeled.

A neural model describing interactions of excitatory and inhibitory neurons, with sufficient structure to couple medication actions, is the Wilson-Cowan model (Wilson and Cowan 1972). This model is interpreted as two interacting populations of cortical neurons where a single variable for each population represents the average spike rate (Figure 1A). The Wilson-Cowan model is mathematically well-understood (Cowan et al. 2016; Bressloff 2010; Benayoun et al. 2010; Buice et al. 2010; Negahbani et al. 2015) with dynamics that can display excitatory bursts and oscillations for different choices of parameters. For the purposes of the current study, we select a parameter range so that the dynamics represent two steady states of spiking activity, a high-rate and low-rate, in two basins separated by a barrier. The hight of the barrier is determined by the parameters of the model and determines the perturbation required to transition from the high-rate state to the low-rate state. The transition from the high-rate state to the low-rate state represents the termination of a cortical activity pattern. If the barrier is high then the system becomes “stuck” in a functional pattern and is interpreted to represent symptoms of catatonia such as postering or perseveration. Parameters of the model determine the synaptic coupling between populations of neurons and internal neural excitability, and these parameters are affected by medications.

In our model, we start with baseline parameter settings with a high barrier to represent catatonia, then calculate the changes in parameters based on the doses of medications in the clinical treatment. We show that the change in the barrier can be correlated with the change in BFCRS score to explain how each medication is impacting symptoms of catatonia. By using the model as a clinical guide to treatment, the clinician can conceptualize the physiological effects of a treatment as controlling cortical excitability to treat catatonia. This allows guidance beyond the safety and efficacy of individual medications to integrate polypharmacy into utilizing additive effects maximize positive outcomes.

## 2 Materials and methods

### 2.1 Data synthesis

Clinical data on BFCRS scores and daily medication dosages were collected and analyzed for clinical treatment purposes. For demonstration purposes, we synthesized surrogate data based on the statistics of the original data set (Conour, 2015). Using the *SVD* (Patki et al. 2016) python package, we constructed a Gaussian copula model based on the daily dosages of medications, and BFCRS scores before and after changes in medication for 12 individuals. The statistical reconstruction method ensured that no personal patient data is present in the published study. The copula model generated many spurious data samples with unrealistic medication doses because there were few individuals included in training the model. To eliminate spurious data, we added rules determined by JC to be unlikely under clinical conditions (see Data Selection Filter, Supplementary Data). The copula model generated 700 subjects and 58 subjects remained after filtering.

### 2.2 Wilson-Cowan model

The pharmaceutical treatments include three classes of medications: anticonvulsants, benzodiazepines, and antipsychotics. These medications operate *via* multiple mechanisms of action, and our approach couples their action to a model of cortical activity. In order to quantify the effects, we developed a two-state model of cortical dynamics that can predict how varying doses affect catatonic symptoms. We use a special case of the Wilson-Cowan equations:

$$\begin{array}{ll} \dot{x_{0}} & =-x_{0}+F_{0}\left(w_{00}x_{0}+w_{01}x_{1}\right)\\ \dot{x_{1}} & =-x_{1}+F_{1}\left(w_{10}x_{0}+w_{11}x_{1}\right) \end{array}$$ (1)

With the spike probability (rate) function:

$$F_{a}\left(x_{a}\right)=\frac{1}{1+\exp\left[-\mu_{a}\left(x_{a}-\theta_{a}\right)\right]}$$ (2)

We interpret *x*_0_ as the average rate of inhibitory interneurons (parvalbumin positive) and *x*_1_ as the average rate of excitatory neurons (cortical pyramidal cells). The parameters of the model were initialized to express three fixed points, one stable fixed point representing a low spike rate, one stable fixed point representing a high spike rate, and a saddle point that is the barrier between the two states. The initial synaptic parameters were chosen to be: *w*_11_ = 8.65, *w*_10_ = 4, *w*_01_ = 13, and *w*_00_ = 9. The parameters for the rate function are *μ*_1_ = 1.2, *θ*_1_ = 2.8, *μ*_0_ = 1.0, and *θ*_0_ = 4.0. We modify these initial parameters to represent the effects of medications in the system, but the effects are small enough to restrict the model behavior to this special case of the Wilson-Cowan model with two stable, and one unstable, fixed points.

A simulation of the Wilson-Cowan equations (Eq. 1) is shown in Figure 1B. The rates are initialized near the high-rate fixed-point (*x*_0_ = 0.6, *x*_1_ = 0.9) and normally distributed (mean = 0 and standard deviation = 0.19) perturbation is injected into the each neural pool at each time-step to simulate noise. The high-rate state is unstable under perturbations and when noise is added, the system will spontaneously transition to the low-rate state. The duration of the time in the high-rate state can be interpreted as a form of working memory (Katori et al. 2011), but here we consider the duration as a phase of activity (Bagi et al. 2022) that can lead to perseveration when the barrier is too high and a large perturbation is required for a state transition. Medications act on parameters of the model to raise or lower the boundary and affect catatonia symptoms.

Figure 1C shows the phase plane for the initial parameters of (Eq. 1). The barrier (*B*) is calculated by a cumulative summation of the excitatory rate gradient $\left(\dot{x_{1}}\right)$ along the *x*_0_-nullcline $\left(N_{I}\right)$ from the high-rate fixed point to the barrier fixed point,

$$B=\underset{n\in N_{I}}{\sum }\dot{x_{1}}\left(n\right)$$ (3)

where the sum is over a lattice of 100 evenly spaced points. The boundary as calculated here is proportional to the minimal perturbation necessary to transition out of the high-rate state basin, and will be compared with BFCRS score.

### 2.3 Coupling treatment doses to model parameters

Clinical doses were converted to changes in the model parameters through a series of calculations. First we approximated the pharmacokinetics of each medication (see Pharmacokinetic Parameters, Supplementary Data) to arrive at a concentration in the cerebrospinal fluid (CSF). Next we calculate the binding to a target, and finally approximate an effect on the model parameters (Spiros et al. 2010; Geerts et al. 2013). The following provides details of the pharmacokinetics and coupling for lamotrigine, lorazepam (and applies to other benzodiazepines according to their affinities), and antipsychotics.

After the effects of medications are calculated, the parameters of the Eq. 1, $\vec{p}=\left[\mu_{0},\mu_{1},\theta_{0},\theta_{1},w_{00},w_{01},w_{10},w_{11}\right]$ are transformed to ${\vec{p}\;}^{\prime }=\left[\mu_{0},\mu_{1}^{\prime },\theta_{0}^{\prime },\theta_{1}^{\prime },w_{00}^{\prime },w_{01}^{\prime },w_{10}^{\prime },w_{11}^{\prime }\right]$ (only *μ*_0_ is unaffected by any medication in this implementation). When the changes in model parameters are calculated, we multiply by an overall factor of $a\cdot {\vec{p}\;}^{\prime }$ , where *a* = 0.35 is an overall medication response factor. This response factor limits the dynamics of the system to maintain two stable fixed points separated by an unstable barrier fixed-point and ensure that the ground state of the system is the low-rate fixed point for all cases. The value of the response factor *a* = 0.35 was found experimentally for the range of doses and combinations of medications in the data. For the personalization demonstration, we replaced this single parameter with an independent value for each individual to calibrate the response to the medications for each subject.

#### 2.3.1 Pharmacokinetics

After patients are admitted for care at Cascadia Behavioral Healthcare, they transition to the treatment over the course of several months. Their BFCRS scores are tested on admittance and after they stabilize on the new treatment, and daily variations in behavior are not measured. Therefore, we base our model on average daily concentrations in the blood and brain to predict the long-term changes in the BFCRS score. To compute the average CSF concentration, *C*_*ave*_, we apply the following function to the clinical daily dose for the synthesized data:

$$C_{\mathrm{ave}}=\frac{F\cdot D}{CL\cdot \tau \cdot K_{p}\cdot M}$$ (4)

where *F* is the bioavailability, *D* is the daily dose (mg), *CL* is the clearance (mg/hr), *τ* is the dose interval (hr), *K*_*p*_ is the brain/blood transport ratio, and *M* is the molecular weight to convert (g/mol) to (nM). A linear response of plasma concentration to dose has been observed in individual patients for two of the medications in treatments (clozapine and lamotrigine), suggesting that the use of linear pharmacokinetics is allowed in our model.

#### 2.3.2 Lamotrigine

There are three targets of lamotrigine in cortical pyramidal cells, the Na^+^-current (Xie et al. 1995), the *I*_*h*_-current (Poolos et al. 2002), and glutamate release (Wang et al. 2001). The first two of these reduce the excitability of pyramidal cells and the third reduces the excitatory output of these neurons. We represent the reduction in excitability in model parameters as an increase in the threshold, *θ*_1_. The reduction in excitatory synaptic out put is represented as a reduction in excitatory weights, *w*_11_ and *w*_10_.

##### 2.3.2.1 Na^+^ *-*current

Lamotrigine reduces Na^+^-current by blocking in Na^+^ channels in pyramidal cells (Xie et al. 1995). We calculate a change in Na^+^-current, *I*_*Na*_, with a binding equation following a calculated lamotrigine concentration, *C*_*LTG*_,

$$\Delta I_{Na}=1-\frac{C_{\mathrm{LTG}}}{{\left(C_{\mathrm{LTG}}+K_{C}\right)}^{n}}$$ (5)

where *K*_*C*_ = 513 uM, *n* = 0.9. To affect the rate in the model, we reduce *θ*_1_ by calculating the effect, *E*_*Na*_ = 1−*p*_*Lam*_ (1−Δ*I*_*Na*_), where *p*_*Lam*_ = 0.15. The reduction in the Na current increases the threshold in excitatory neurons by multiplicative factor, $\theta_{1}^{\prime }=\theta_{1}/E_{Na}$ , where the prime indicates the modified parameter.

##### 2.3.2.2 *I*_*h*_ *-*current

Lamotrigine shifts the I-V activation curve of the *I*_*h*_ current (Poolos et al. 2002) and decreased evoked firing rate, Δ*x*_1_ = 1–0.004*C_*LTG*_, for Δ*x*_1_ > 0 and where *C*_*LTG*_ is the average concentration of lamotrigine in CSF. To represent this effect in our model parameters, we modify the threshold, *θ*_1_, in pyramidal cells. The shift in based on the spike probability function linearized near threshold *F*_1_ (*x*_1_) = 1/2 + (*μ*_1_/4) (*x*_1_ − *θ*_1_), so that *θ*_1_ will be increased by the effect, *E*_*h*_ = 1 − *p*_*Lam*_ (1 − Δ*x*_1_), where *p*_*Lam*_ = 0.15. The reduction in the Na current increases the threshold in excitatory neurons by, $\theta_{1}^{\prime }=\theta_{1}/E_{h}$ .

##### 2.3.2.3 Glutamate release

Lamotrigine reduces glutamate release from excitatory synapses proportionally to the concentration *C*_*LTG*_ (Wang et al. (2001)), Δ*G* = 1–0.004*C_*LTG*_ for Δ*G* > 0. The excitatory synaptic parameters, *w*_11_ and *w*_10_, are affected by the effect, *E*_*glu*_ = 1 − *p*_*Lam*_ (1 − Δ*G*), where *p*_*Lam*_ = 0.15. The reduction in the Glutamate release decreases the excitatory synaptic parameters by, $w_{11}^{\prime }=w_{11}E_{\mathrm{glu}}$ and $w_{10}^{\prime }=w_{10}E_{\mathrm{glu}}$ .

#### 2.3.3 Benzodiazepines

Benzodiazepines such as lorazepam increase GABA_*A*_ currents following binding at the BZD receptor site. The increase in GABA_*A*_ synaptic current is represented in the model as an increase in the inhibitory synaptic weights, *w*_01_ and *w*_00_. To calculate the receptor occupation we follow results reported in (Miller et al. 1987):

$$R_{\mathrm{BZD}}=\frac{{\left(C_{\mathrm{Lor}}\right)}^{A}}{{\left(C_{\mathrm{Lor}}\right)}^{A}+B}$$ (6)

where *A* = 1.4328, *B* = 73.89 (ng/gm), and *C*_*Lor*_ is the average concentration of lorazepam in CSF. The inhibitory synaptic parameters, *w*_01_ and *w*_00_, are modified in the model by increasing the inhibitory synaptic parameters proportionally to the receptor occupation, $\Delta w_{11}^{\prime }=w_{11}\left(1+R_{\mathrm{BZD}}\right)$ and $w_{10}^{\prime }=w_{10}\left(1+R_{\mathrm{BZD}}\right)$ . All other benzodiazepines are treated in the same manner to increase inhibitory synaptic parameters.

#### 2.3.4 Antipsychotics

These medications bind competitively with endogenous neurotransmitters to specific receptors. We use an exact form of the competitive binding formula (Wang 1995):

$$\begin{array}{ll} a & =K_{A}+K_{B}+C_{A}+C_{B}-1\\ b & =K_{B}\left(C_{A}-1\right)+K_{A}\left(C_{B}-1\right)+K_{A}K_{B}\\ c & =-K_{A}K_{B}\\ \delta & =\arccos\left(\frac{-2a^{3}+9ab-27c}{2\sqrt{{\left(a^{2}-3b\right)}^{3}}}\right)\\ R_{oc} & =C_{A}\frac{2\sqrt{a^{2}-3b}\cos\left(\theta /3\right)-a}{3K_{A}+\left(2\sqrt{a^{2}-3b}\cos\left(\theta /3\right)-a\right)} \end{array}$$ (7)

where *K*_*A*_ is the binding affinity of the endogenous neurotransmitter, *C*_*A*_ is the average concentration of the endogenous neurotransmitter, *K*_*B*_ is the binding affinity of the medication, and *C*_*B*_ is the average concentration of the medication. *R*_*oc*_ is the receptor occupation by the endogenous neurotransmitter and will be used to estimate the activation level of the receptor. In this study, endogenous levels of neurotransmitters were dopamine (tonic) = 37 mM, dopamine (burst) = 200 mM, serotonin = 3.9 mM, and acetylcholine = 10 mM (Dreyer et al. 2010; Paterson et al. 2010).

##### 2.3.4.1 D1 activation effect

The endogenous concentration at dopamine synapses depend on the firing pattering so that simulations estimate (Dreyer et al. 2010) that tonic activity yields 37 ± 1.2 nM and bursts yield 100–300 nM. According to data in (Lapish et al. 2007), D1 activation decreases the slope parameter (*μ*_1_) of the rate function in excitatory neurons, $\mu_{1}^{\prime }=\mu_{1}\left(1-\left(R_{oc}-R_{\mathrm{con}}\right)/R_{\mathrm{con}}\right)$ , where *R*_*con*_ is the control level. D1 activation decreases the threshold (*θ*_0_) in inhibitory interneurons, $\theta_{0}^{\prime }=\theta_{0}\left(1-\left(R_{oc}-R_{\mathrm{con}}\right)/R_{\mathrm{con}}\right)$ . D1 activation increases *w*_11_, and *w*_10_ because at low concentrations ( < 50 uM) by acting preferentially on D1-like receptors to increase NMDA receptor-mediated transmission (Lee et al. 2002), and increases *w*_01_, that we represent by $w_{ab}^{\prime }=w_{ab}\left(1+\left(R_{oc}-R_{\mathrm{con}}\right)/R_{\mathrm{con}}\right)$ where (*a*, *b*) = (1, 1), (1, 0), and (0, 1).

##### 2.3.4.2 D2 activation effect

At high concentrations (≥100 uM) DA activates D2-like receptors and suppress NMDA function (Kotecha et al. 2002), that we represent by decreasing *w*_11_ and *w*_10_, that we represent by $w_{ab}^{\prime }=w_{ab}\left(1-\left(R_{oc}-R_{\mathrm{con}}\right)/R_{\mathrm{con}}\right)$ where (*a*, *b*) = (1, 1) and (1, 0). D2 also Increases the slope parameter (*μ*_1_) of probability function in excitatory neurons (pyramidal cells (Lapish et al. 2007), $\mu_{1}^{\prime }=\mu_{1}\left(1+\left(R_{oc}-R_{\mathrm{con}}\right)/R_{\mathrm{con}}\right)$ .

##### 2.3.4.3 5-HT1A activation effect

The effect of 5-HT1A receptor activation has been found to increase the spike threshold in excitatory neurons (pyramidal cells, (Foehring 1996), and we model the effect as a linear increase in the threshold of excitatory neurons, $\theta_{1}^{\prime }=\theta_{1}\left(1+\left(R_{oc}-R_{\mathrm{con}}\right)/R_{\mathrm{con}}\right)$ .

##### 2.3.4.4 5-HT2A activation effect

The effect of 5-HT2A receptor activation has been found to decreases the spike threshold in excitatory neurons (pyramidal cells, (Carr et al. 2002), and we model the effect as a linear decrease in the threshold of excitatory neurons, $\theta_{1}^{\prime }=\theta_{1}\left(1-\left(R_{oc}-R_{\mathrm{con}}\right)/R_{\mathrm{con}}\right)$ .

##### 2.3.4.5 M1 activation effect

The effect of M1 receptor activation has been found to decreases the spike threshold in excitatory neurons (pyramidal cells) (Perez-Rosello et al. 2005), and we model the effect as a linear decrease in the threshold of excitatory neurons, $\theta_{1}^{\prime }=\theta_{1}\left(1-\left(R_{oc}-R_{\mathrm{con}}\right)/R_{\mathrm{con}}\right)$ .

### 2.4 Statistical analysis

Statistical analysis was performed on simulated data and model results using python SciPy v1.5.4 statistical functions (Virtanen et al. 2020) and by direct calculations. The effect sizes comparing before and after treatment were calculated as the difference between means of the two groups divided by a standard deviation for the data. The associated p-value is calculated with the one-way ANOVA test. The relationship between the clinical BFCRS scores and the barrier in the model was measured with the Pearson correlation (*r*) using a linear regression analysis.

## 3 Results

### 3.1 Synthesized data

**FIGURE 2:**
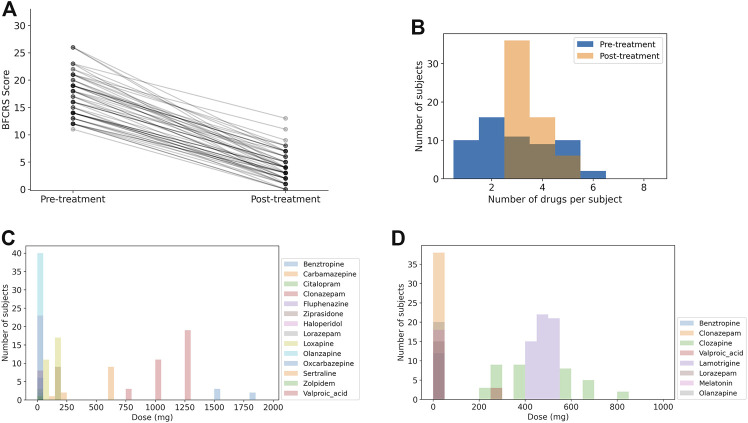
Summary of synthesized data. Note that the number of medications and distributions of doses is more restricted after treatment has stabilized. **(A)** BFCRS score for 58 synthesized data subjects before and after treatment. **(B)** Distribution of medication doses across all subjects before treatment. **(C)** Distribution of medication doses across all subjects after treatment. **(D)** Distribution of the number of medications for each subject before and after treatment.

A summary of the synthesized dataset used in this study is shown in Figure 2, and the statistics of the medication combinations and dose ranges are consistent with the clinical patient data set. The mean BFCRS score before treatment is 17.3 ± 3.9 (std) and after treatment is 4.1 ± 2.8, resulting in an effect size of 2.7 (p $<10^{-20}$ ) for the treatment (Figure 2A). The treatment results in a reduction in the BFCRS score for all subjects, with a minimum reduction of 9, and a maximum of 23.

In Figure 2B we show a histogram of the number of medications for each subject to demonstrate that the patients transition from a broad range of care to a more limited set of medications. The distribution of doses for medications upon admission (pre-treatment) and following stabilization of the treatment (post-treatment) are shown in Figures 2C,D. Again, we see that the diversity of medications is reduced to focus treatment on catatonia symptoms with the minimal set of medications to simplify care.

### 3.2 Dose sensitivity

**FIGURE 3:**
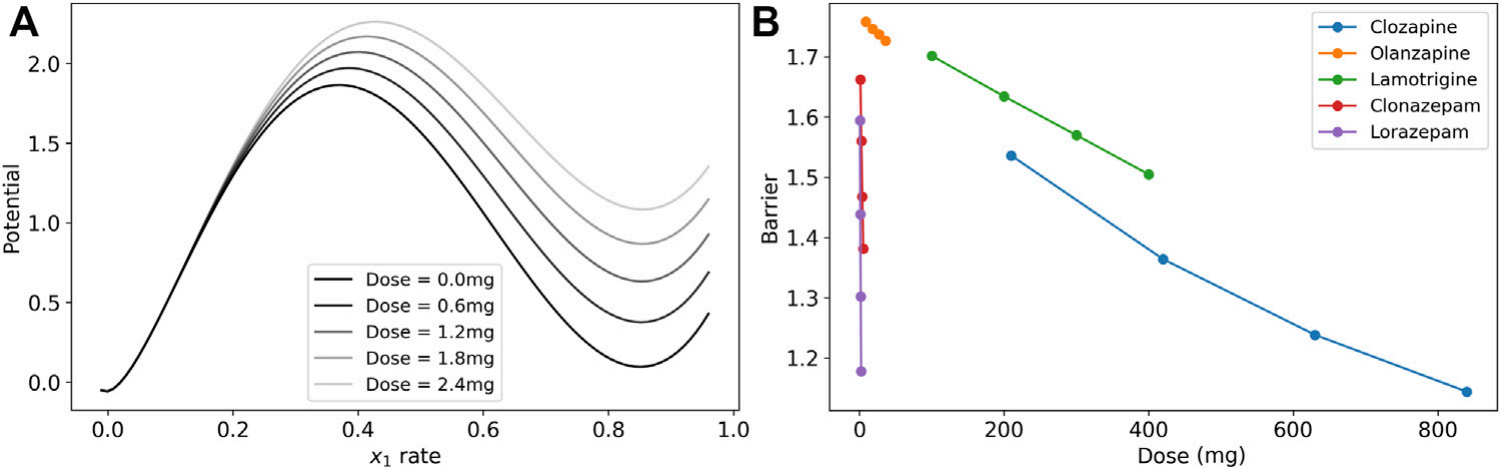
Dose response of the model barrier to medications. **(A)** Dose response of potential function (line integral of $\dot{x_{1}}$ in Eq. 1 on the *x*_0_-nullcline) to show how the barrier becomes smaller with increasing doses of lorazepam. The two stable fixed points are where the excitatory rate is 0 and ∼0.85. The peak of the barrier is the unstable fixed point where the excitatory rate is ∼0.4. The vertical distance from the high-rate basin to the unstable peak is the barrier. **(B)** Dose response of model parameters for clozapine, olanzapine, lamotrigine, clonazepam, and lorazepam. The barrier between the high-rate state and the low-rate state for these medications is reduced in the treatment.

We visualize the change in the barrier in Figure 3A for the case of lorazepam. At the dose = 0.0 mg (black line), there are two minima in the potential of the model where the excitatory rate is zero and near 90%. The maximum near 40% is the unstable fixed point that is the boundary between the two states. As the dose increases (lighter gray lines) the depth of the higher-rate state decreases at a faster rate than the height of the unstable fixed point, and the depth of the potential well is reduced. This reduction in the depth (reduction of the boundary) is interpreted as a reduction of symptoms of catatonia because patients are less likely to become stuck in particular high-rate activity patterns.

To illustrate the effects of each medication in the post-treatment cases, we calculated the model barrier height across the range of doses from the clinical data and tested the model for the change in the barrier for lamotrigine, two benzodiazepines, and two antipsychotics (Figure 3B). Each of the medication in the figure reduced the barrier in a nearly linear dose response in this range as demonstrated by a linear regression analysis that finds that *r* ^2^ > 0.99 (p < 0.01) in all cases except clozapine where *r* ^2^ > 0.98 (p < 0.01).

The three classes of medications, lamotrigine, benzodiazepines, and antipsychotics, affect the system through different mechanisms of action. Lamotrigine acts to reduce excitation by both reducing the excitability of the excitatory neuron population and reducing the excitatory synaptic weights. The benzodiazepines act through increasing the inhibitory synaptic weights to reduce the boundary between states.

The antipsychotics have more complicated mechanisms of action through dopamine, serotonin, and muscarinic receptors. We model two types of dopamine receptors, D1 and D2. In our model, D1 receptor activation decrease the excitability of the excitatory neuron population and increase the excitability of the inhibitory neuron population, both contributing to increasing barrier when D1 receptors are blocked by antipsychotics. However, D1 activation also increases excitatory synaptic transmission to have the opposite effect on the barrier by antipsychotics that block D1. The D2 receptor activation reduces excitatory synaptic transmission and increases the excitability of the excitatory neuron population leading to opposite effects. Activation of the two serotonin receptors included the model (5-HT1A and 5-HT2A) have opposite effects on the excitability of the excitatory neuron population, and M1 receptor activation increases their excitability. The affect of each antipsychotic depends on the affinity of the molecule to each receptor in competition with the background level of neurotransmitter, and we find that there is a net decrease in the barrier for increasing dose of both clozapine and olanizapine. Clozapine has a more mixed effect on several parameters with the largest effect on the threshold of excitatory neuron that reduces their overall excitability.

**FIGURE 4:**
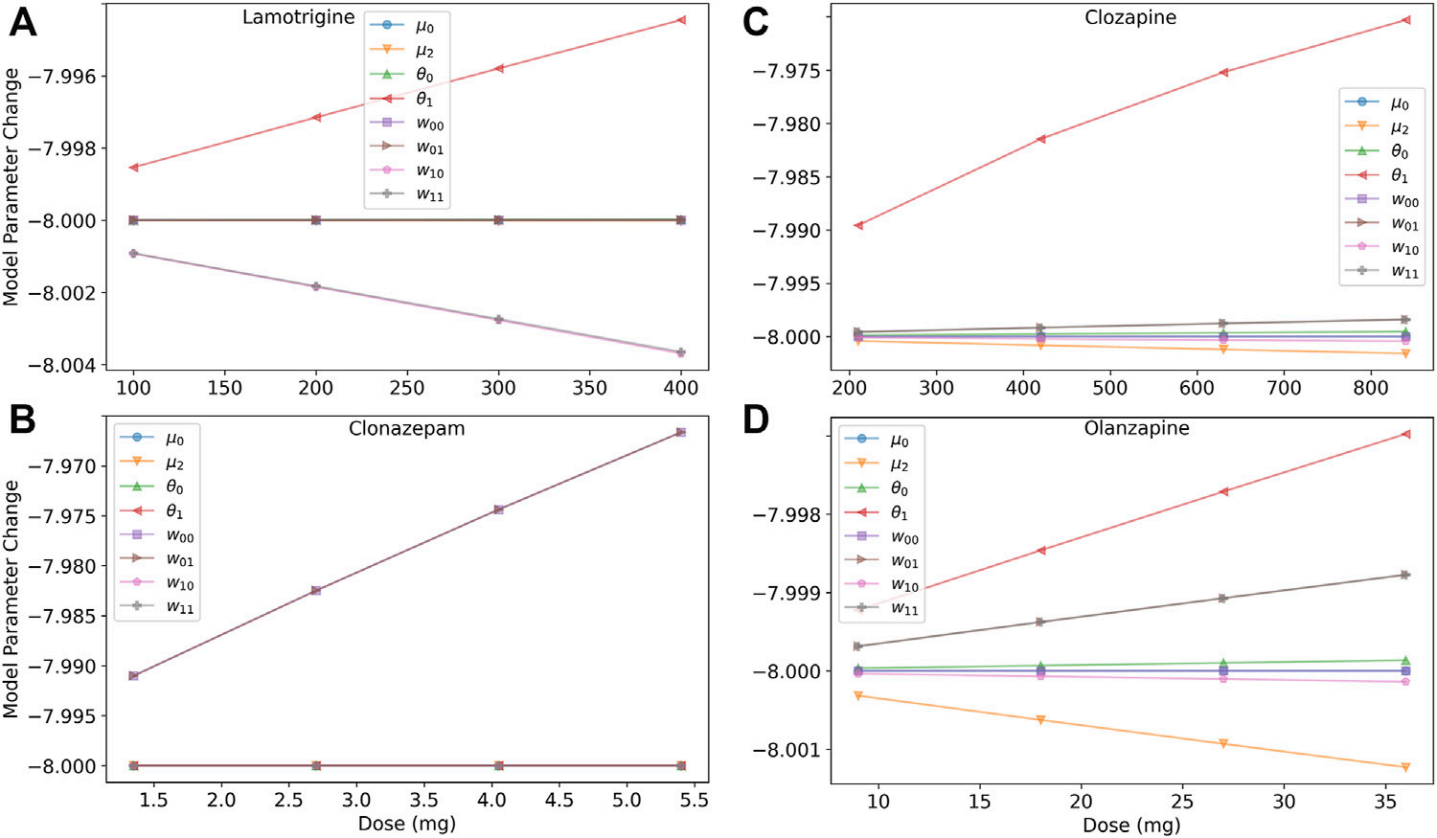
Dose response of model parameters for lamotrigine, clonazepam, clozapine, and olanzapine. **(A)** The dose response of the model’s parameters for lamotrigine shows that the threshold of excitatory neurons (*θ*_1_) increases with increasing dose leading to a decrease of the neurons’ excitability. The excitatory synaptic parameters (*w*_11_ and *w*_10_) decrease leading to a reduced excitation of the system. **(B)** The dose response of the model’s parameters for clozapine shows that several parameters are affected, but the largest effect is an increase of the threshold in excitatory neurons (*θ*_1_) due to blocking M1 and 5-HT2A receptors reducing the excitation of the system. **(C)** The dose response of the model’s parameters for olanzapine shows less of a an increase in the threshold in excitatory neurons than clozapine, and a finer scale view of the other model parameters. **(D)** The dose response of the model’s parameters for clonazepam show that only the inhibitory synaptic parameters are affected.

To help untangle the competing effects of the medications, we investigated the dose response of model parameters, as shown in Figure 4. Lamotrigine reduces excitability of excitatory neurons through the threshold by increasing *θ*_1_, and reduces the excitatory synaptic weights, *w*_10_ and *w*_11_ (Figure 4A) The benzodiazepine (clonazepam, Figure 4B) has the simplest action and affects only the inhibitory synaptic weights (*w*_00_ and *w*_01_) in the model. The increased inhibition in the system reduced the overall excitability, weakening the high-rate state and reducing the boundary. The antipsychotics affect multiple parameters (Figures 4C,D), but the cumulative effect is to reduce barrier height. Clozapine has a stronger effect on the threshold *θ*_1_ than olanzapine leading to a greater reduction of the barrier.

### 3.3 BFCRS clinical scale

**FIGURE 5:**
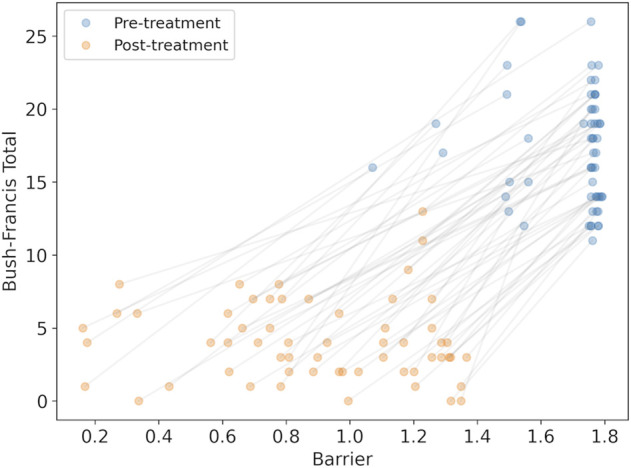
The synthesized BFCRS score *versus* the barrier calculated by the model for subjects before and after treatment. The grey lines associate the pre- and post-treatment scores for the same subject.

We calculated the changes in the model parameter for each synthesized subject caused by medications at admission, and then after treatment was stabilized. With the modified parameters we could calculated the barrier between the high-rate state and the low-rate state to observe whether the barrier was reduced. A reduction in the barrier is interpreted as an improvement in catatonic symptoms. We find that the barrier was reduced in all cases (mean reduction 0.80 ± 0.32, with minimum reduction of 0.19), consistent with clinical observations. We could then compare the BFCRS clinical score with the barrier to visualize the effect of the treatment (Figure 5).

There is a clear reduction in the barrier (effect size = 2.14, p $<10^{-20}$ ), consistent with the reduction in BFCRS score. However, there appears to be poor individual prediction by the model as observed in the even distribution of the changes across the subjects before and after treatment in Figure 5, and a linear regression results in *r* ^2^ = 0.53. To test the reliability of the model in predicting changes in individual cases, we compared the change in BFCRS score and barrier and found a correlation of *r* ^2^ = 0.11 (p <0.01 ). We have confirmed that this is not due to lost correlations in our synthesized data, and may be attributed to individual differences between subjects in both their pre-treatment disease state and their response to the medications.

### 3.4 Combination efficacy

**FIGURE 6:**
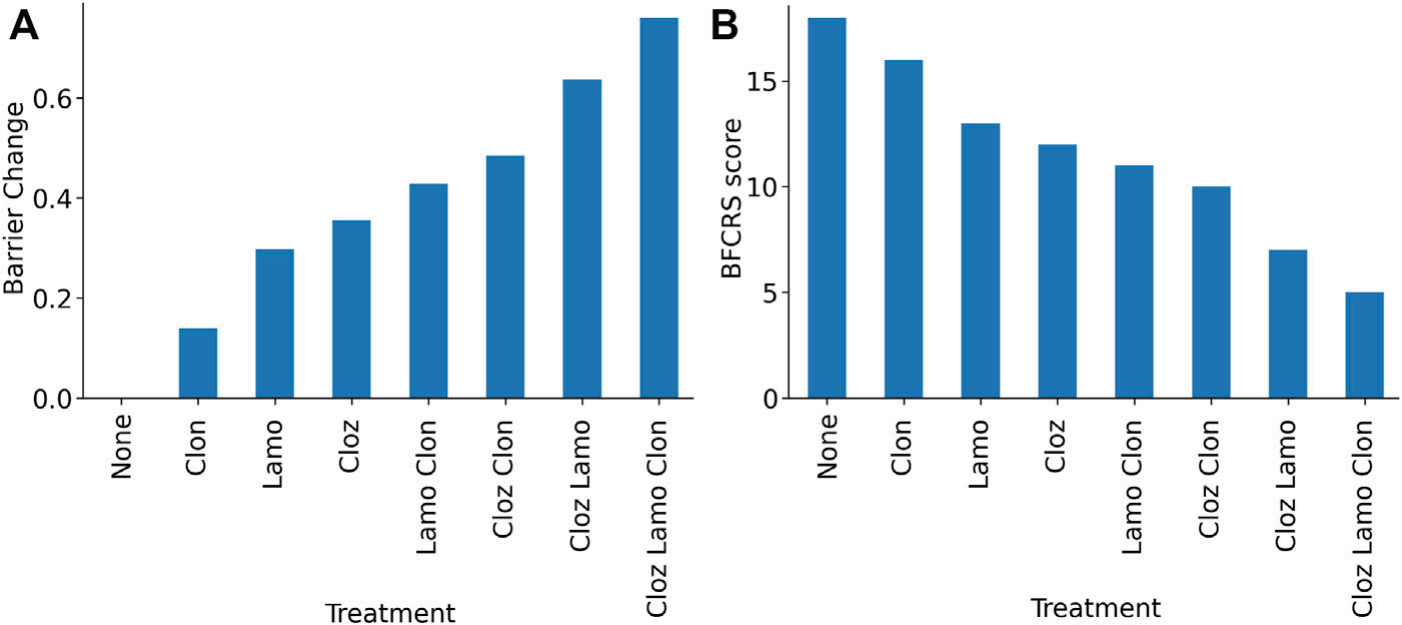
Model results for combinations of lamotrigine, clozapine, and clonazepam demonstrating the additive effects. **(A)** Barrier for combinations of medications in the treatment protocol. **(B)** Predicted BFCRS for combinations of medications in the treatment protocol.

The combination of medications in the treatment has been clinically observed to be additive, and this observation can be explained by the parallel mechanisms of action. Lamotrigine and the benzodiazepines act on different sites, excitatory neurons and inhibitory synapses. Although the antipsychotics have some overlap with these parameters in the model, they act through different receptors. In the dynamic range of medication effects on the barrier size, the dose response is nearly linear, and we find an additive effect of the combination (Figure 6A). To relate the effect back to the clinic, we can use a linear mapping between the BFCRS score and the boundary to interpret the boundary as a BFCRS score and predict the effect of each medication and their combinations on the average subject. We calculate the mean BFCRS score and mean barrier for the population, before and after the treatment to obtain the mapping, and then plot the BFCRS score in Figure 6B.

### 3.5 Personalization

The model is good at predicting large changes in BFCRS score for the population as a whole, but more exact predictions of individuals should be possible with further parametrization. Ultimately, the model could then be used as a tool for informing clinical care and refining treatments. Because the model has few parameters to tune, then each subject could have a personalized model for use in the clinic. We personalized the model by calibrating the initial state with model parameters, and then adjusted the dose response parameter for each individual subject.

The first adjustment was to tune individual Wilson-Cowan model based on the initial BFCRS score for each patient. The barrier size can be adjusted in the Wilson-Cowan model so that patients with high BFCRS scores will have a corresponding model with a high barrier. We have attempted to tune the *w*_01_ model parameter to this end, but no clear result could be seen in the correlation of the outcomes to treatment. Further research will be needed to determine whether different model parameters need to be tuned to be more representative of the pathology underlying catatonia.

The second adjustment was to calibrate the individual dose response with model coupling parameters to the effect on BFCRS score. As patients are admitted to the residence, they transition their medication to the new regimen, and measures of the BFCRS score inform how each individual is affected by removing and adding medications. These changes in BFCRS score could be used to calibrate individual mechanisms and how they couple to model parameters. Such a tuning could create a model that will adapt along with the patient, and improves in its prediction power over time.

**FIGURE 7:**
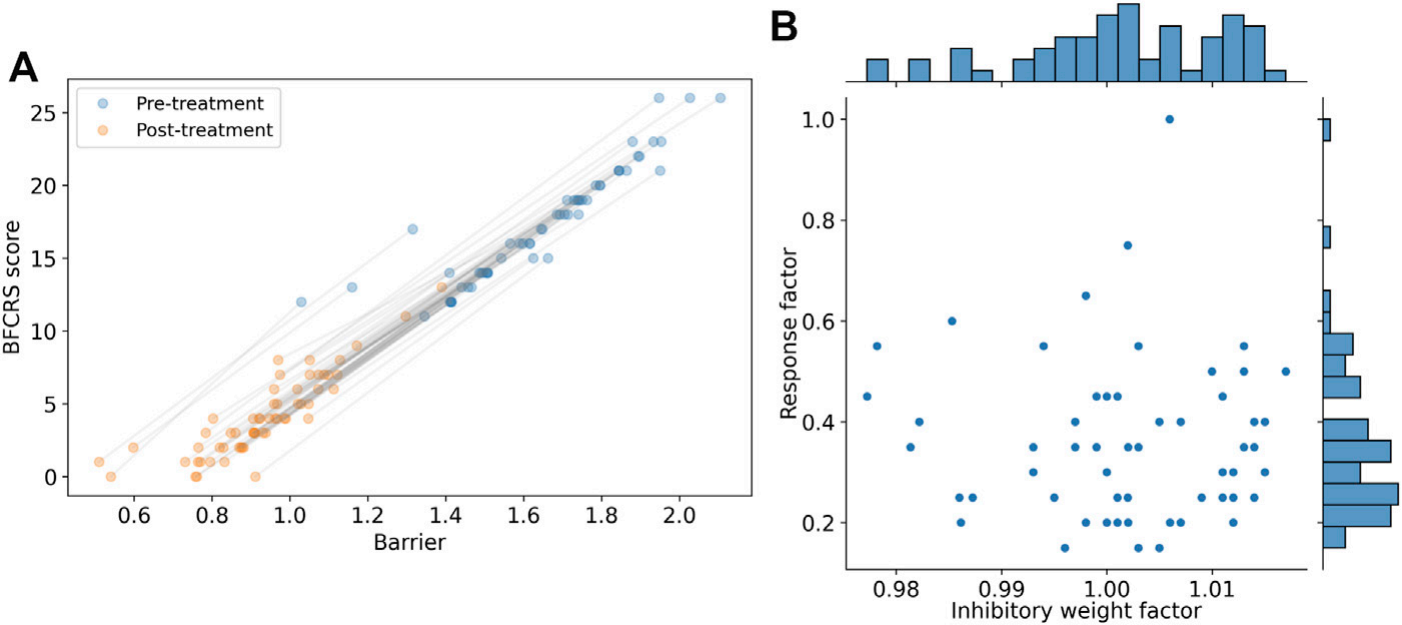
Demonstration of personalization potential of the model for individualized clinical predictions. **(A)** Personalized model prediction of barrier and the synthesized BFCRS score for subjects before and after treatment. The grey lines associate the pre- and post-treatment scores for the same subject. **(B)** Scatter plot and histograms of the parameters used for personalization (Inhibitory weight factor to modify *w*_00_ and the medication response factor). The scatter plot reveals no significant correlation between the personalization parameters (*r* ^2^ = 0.001 and p >0.7 ).

The results of these two modification are shown in Figure 7 where the new prediction of the barrier is compared with the BFCRS score. We find a higher correlation between the model barrier and the clinical score (*r* ^2^ = 0.97) and our comparison of the change in BFCRS score and barrier across individual yields a correlation of *r* ^2^ = 0.92 (p $<10^{-30}$ ). These results give confidence that the effects predicted by the model can guide further changes in medication, and aid the psychiatrist in clinical decisions.

## 4 Discussion

The objective of this study was to demonstrate that a simple cortical model, with excitatory and inhibitory neural populations, is sufficiently descriptive to explain and predict clinical outcomes in schizophrenia patients with catatonia. The pharmaceutical coupling of the treatments to model parameters are based on known mechanisms of action in cortical neurons: pyramidal cells and parvalbumin positive inhibitory interneurons. We have demonstrated the utility of the model for explaining the observed clinical outcomes by tracing the action of medications to changes in the model dynamics by interpreting the change in the barrier between states as a change in a clinical measure, the BFCRS score. The model supports the clinical observation that the 3-medication combination, clozapine, lamotrigine, and a benzodiazepine, is additive, and explains how the pathways of action are independent on a mechanistic level. Finally, we took a first step at personalization of the model for individual subjects, with the goal of supporting individual clinical decisions with mechanistic explanations.

Augmenting psychiatric practice with a simple mechanistic model encourages a conceptual shift to a focus on reducing cortical excitability, either through reducing excitability of pyramidal neurons, or increasing inhibition. Each of the three medications are optimized on their own for safety and efficacy, but since they act on the excitability of the system through different mechanisms, they can have an additive effect on catatonic symptoms. Further use of this approach can suggest other means of controlling cortical excitability and inspire new treatment protocols.

Conceptualizing the action of this treatment as modifying excitability and connectivity of neuron populations also suggests mechanisms of observed clinical improvements. The clinical observation that reduced chronic catatonic features lead to meaningful improvements in social and cognitive function suggests that reducing the barrier represents a physical improvement in brain network connectivity and dynamical processing. Bursts of neural activity that control behavioral patterns become more flexible with a reduced barrier between states of excitation, and that flexibility leads to more fluid cognitive function and social behavior.

### 4.1 Extensions of the model

The model is based on cortical circuitry, in part because catatonia is thought to have a cortical origin. However, antipsychotics also target the striatum. Extending the model to include a cortical-striatum-thalamic loop would include additional dynamics that are presently missing. As yet, it is unknown if such an extension will add a precision that is visible in clinical usage, but this would be a rich area to explore.

One avenue to improve the model’s predictions is to further personalize the model by individualizing the pharmacokinetics for each patient. When clozapine is administered, safety considerations require blood samples, and blood levels of clozapine have been recorded from many patients in this cohort. There is a wide variation in the dose response to blood serum concentration of clozapine, and these variations are not currently included in the model. We have tested the robustness of our results to ensure this observed variance does not affect the conclusions in this study, but clearly such an addition to the model will help to refine individual cases.

Clinically the BFCRS score can be low in lower functioning individuals and high in higher functioning individuals. There may be a correlation with changes in score and functional status for a population, but it is not yet clear with individual cases. Big changes can lead to little benefit sometimes, small changes can lead to large benefits. Additional neural circuitry in the model, such as a striatal-thalamic loop may help to explain a separation between BFCRS score and overall function. Such modification could be aided by analysis with a larger subject pool that may help to discern subgroups in responses to treatment.

Further clinical variables may provide new insights into how model outputs can be interpreted. Although the BFCRS score has provided a good clinical guidance for this cohort, the addition of either cognitive or motor measures could augment the model’s interpretation. Furthermore, additional clinical measures could add constraints that require a more detailed model, such as adding a striatum, that the BFCRS score alone will not capture. Although further complications may degrade the causal interpretability because of added complex dynamics, there are likely parameter regions with simpler dynamics that may broaden the applicability to other symptoms of psychiatric disease.

## Data Availability

The datasets generated for this study can be found in the repo for Cascadia-Behavioral-Healthcare: https://github.com/pdroberts/cascadia-behavioral-healthcare.git.
