# Supplementary material for: Mechanistic modeling as an explanatory tool for clinical treatment of chronic catatonia
Source: Front Pharmacol. 2022 Nov 9;13:1025417. doi: 10.3389/fphar.2022.1025417 (PMC9682077; doi:10.3389/fphar.2022.1025417)
Supplement: Supplementary file 1 [file DataSheet1.pdf]

Supplementary Data

Mechanistic modeling as an explanatory tool for clinical treatment of chronic catatonia
Roberts and Conour (2022) *Frontiers Pharmacology*

Data Selection Filter: *After synthetic data was generated, the following sequence of filters were implemented to remove subjects that did not conform to clinical practice or observations.*
Remove subjects if pre-treatment includes clozapine.
Remove subjects if pre-treatment includes lamotrigine.
Remove subjects if pre-treatment has olanzapine is greater than 20mg/d.
Remove subjects with pre-treatment includes both clozapine and olanzapine.
Remove subjects if post-treatment of Lamotrigine is less than 400mg of greater than 500mg.
Remove subjects if pre-treatment does not include antipsychotics.
Remove subjects if post-treatment does not include antipsychotics.
Remove subjects if post-treatment includes methylphenidate.
Remove subjects if pre-treatment BFCRS score is less than or equal to post-treatment score.
Remove subjects if their BFCRS score changes less than 9 (for consistency with clinical dataset).
Remove subjects if the number of post-treatment medications is less than 3.
Remove subjects if post-treatment includes both clonazepam and lorazepam.
Remove subjects if pre-treatment has more than 1 mood stabilizer.
Balanced the number of subjects with clozapine post-treatment to 80%.

Pharmacokinetic Parameters: Partial list of the most prominent medications in the study.

param	Clozapine	Olanzapine	Lamotrigine	Clonazepam	Lorazepam
bioavailability	0.65	0.87	0.950	0.9	0.85
clearance (L/hr)	31.00	21.80	2.100	2.1	4.30
blood/brain ratio	1.00	1.00	1.900	1.0	1.53
mole wt (g/mol)	326.82	312.43	256.091	315.7	321.20

Receptor Affinities: Partial list of the most prominent medications in the study from (Roth and Lopez 2006).

receptors	Dopamine	5-HT	Clozapine	Olanzapine	Lamotrigine	Clonazepam	Lorazepam
D1	130.0	9690.0	89.0	25.00	10000	10000	10000
D2	469.43	10000	28.0	3.00	10000	10000	10000
5-HT1A	8248.0	2.789	104.8	610.00	10000	10000	10000
5-HT2A	10000	20.80	1.0	1.48	10000	10000	10000
M1	10000	10000	1.4	2.00	10000	10000	10000

Roth, B., and Lopez, E. (2006). Psychoactive drug screening program Ki database.
Available at: https://pdsp.unc.edu/databases/kidb.php.
